# Dementia Prevalence and *Onchocerca volvulus* Infection among Rural Elderly Persons in the Ntui Health District, Cameroon: A Population-Based Study

**DOI:** 10.3390/pathogens13070568

**Published:** 2024-07-06

**Authors:** Wepnyu Yembe Njamnshi, Joseph Nelson Siewe Fodjo, Kongnyu Gamnsi Njamnshi, Leonard Ngarka, Michel K. Mengnjo, Leonard N. Nfor, Martine A. F. Tsasse, Julius N. Taryunyu Njamnshi, Gladys Maestre, Jose E. Cavazos, Sudha Seshadri, Laurent S. Etoundi Ngoa, Marie-Thérèse Obama Abena Ondoa, Bernard Fongang, Anne-Cécile Zoung-Kanyi Bissek, Alfred K. Njamnshi

**Affiliations:** 1 Brain Research Africa Initiative (BRAIN), Yaoundé P.O. Box 25625, Cameroon; wepnyu.njamnshi@brainafrica.org (W.Y.N.); kongnyu.njamnshi@brainafrica.org (K.G.N.); lngarka@yahoo.com (L.N.); mengnjomichel@yahoo.com (M.K.M.); nforleo@yahoo.com (L.N.N.); ntnjulius@yahoo.com (J.N.T.N.); fongang@uthscsa.edu (B.F.); annezkbissek@yahoo.fr (A.-C.Z.-K.B.); alfred.njamnshi@brainafrica.org (A.K.N.); 2 Neuroscience Lab, Faculty of Medicine and Biomedical Sciences, The University of Yaoundé I, Yaoundé P.O. Box 1364, Cameroon; 3 Division of Health Operations Research, Ministry of Public Health, Yaoundé P.O. Box 1937, Cameroon; 4 Global Health Institute, University of Antwerp, Doornstraat 331, 2610 Antwerp, Belgium; 5 Department of Neurology, Central Hospital Yaoundé, Yaoundé P.O. Box 25625, Cameroon; 6 Higher Institute for Scientific and Medical Research, Yaoundé P.O. Box 5797, Cameroon; augustaflore@yahoo.fr; 7 Department of Neurosciences, University of Texas Rio Grande Valley School of Medicine, Brownsville, TX 78520, USA; gladys.maestre@utrgv.edu; 8 Glenn Biggs Institute for Alzheimer’s & Neurodegenerative Diseases, UT Health San Antonio, 7703 Floyd Curl Drive, San Antonio, TX 78229, USA; cavazosj@uthscsa.edu (J.E.C.); seshadri@uthscsa.edu (S.S.); 9 Department of Neurology, UT Health San Antonio, 7703 Floyd Curl Drive, San Antonio, TX 78229, USA; 10 Department of Neurology, Boston University School of Medicine, Boston, MA 02118, USA; 11 Department of Population Health Sciences, University of Texas Health Science Center, San Antonio, TX 77030, USA; 12 Department of Biochemistry and Structural Biology, University of Texas Health Science Center, San Antonio, TX 77030, USA; 13 Brain Research Africa Initiative (BRAIN), 1226 Geneva, Switzerland

**Keywords:** dementia, CSID, elderly, onchocerciasis, Cameroon

## Abstract

Recent research suggests that infection with *Onchocerca volvulus* induces neurocognitive decline. This study sought to compare the cognitive outcomes of elderly persons based on onchocerciasis infection status and report the overall prevalence of dementia in the rural Ntui Health District in Cameroon. A community-based approach was used to recruit 103 participants aged ≥60 years. Dementia screening was done using the Community Screening Interview for Dementia (CSID) tool with a cut-off value of ≤29.5. *O. volvulus* infection was determined via microscopic examination of skin snips and serological testing of Ov16 antibodies using rapid diagnostic tests. Overall, the prevalence of dementia was 10.7%. Among the tested individuals, 17.9% (15/84) and 62.1% (41/66) were positive for *O. volvulus* and Ov16 antibodies, respectively. A multivariable linear regression model of CSID scores found a significant positive association with education level (8.654; 95% CI: 2.0870 to 15.222). However, having a positive skin snip for *O. volvulus* (−3.399; 95% CI: −6.805 to 0.007) and inhaling tobacco (−5.441; 95% CI: −9.137 to −1.744) tended to lower the CSID scores. Ongoing onchocerciasis transmission in the Ntui Health District may constitute a risk factor for dementia. Strengthening onchocerciasis elimination and adopting healthier lifestyles would contribute to dementia prevention among the elderly residing in endemic communities.

## 1. Introduction

The sub-Saharan Africa (SSA) region comprises 48 countries and contains about 1.18 billion residents, with an annual population growth of 2.6%. With improvements in healthcare provision and research, life expectancy has been on the rise globally and particularly in SSA, where it has experienced a 10-year increase from 51 years in 2000 to 61 years in 2022 [1]. According to the World Health Organization (WHO) estimates, by 2030, one in six people in the world will be aged 60 years and over [2]. The fact that people live longer is a major driving force behind the observed demographic shift towards older populations, a situation referred to as population aging. Although population aging initially concerned only high-income countries (HICs), low- and middle-income countries (LMICs) are increasingly confronted with huge demographic transitions as well. At the current rate, LMICs are expected to host two-thirds of the global population of people aged over 60 years by the year 2050 [2].

A consequence of population aging is the increasing number of persons susceptible to geriatric conditions. For instance, the incidence of dementia (characterized by memory loss and one or more other neurological alterations that interfere with social and occupational living) has been shown to increase steadily after the age of 60 years [3]. Current epidemiological trends suggest that the global prevalence of dementia might double every 20 years, increasing from 35.6 million people in 2010 to 115.4 million in 2050. Zooming in on SSA, a recent meta-analytic study estimated the age-standardized prevalence of dementia in people aged ≥60 years at 6.4% [4]. It was further shown that the prevalence of dementia increased exponentially with age, doubling with every 7.2-year increment in age; considering both sexes, dementia prevalence rose from 1.7% at 60–64 years to 23.3% among the 85+ individuals. In addition to dementia, another clinical entity worth mentioning is cognitive impairment (CI), which refers to the progressive decline of some cognitive functions and constitutes a transitory phase between normal aging and dementia [5]. The 2022 symposium on dementia and brain aging in LMICs in Nairobi highlighted that two-thirds of persons with dementia live in LMICs, where the prevalence is expected to more than double, while research and costs are skewed towards HICs [6]. The Nairobi Declaration from this symposium called on global action to improve dementia research, care, and policy through national action plans in LMICs [7].

There is a paucity of data on dementia in Cameroon, with only one population or community-based study investigating cognitive impairment among the elderly [8]. Using the mini-mental state exam, the study found that 33.3% of rural elderly Cameroonians (≥50 years) were cognitively impaired [8]. In the wider Central African region to which Cameroon belongs, community-based studies conducted in the Congo and the Central African Republic revealed that the prevalence of dementia among ≥65-year-olds ranged from 6.7% to 8.1%, while 28.5% to 37.9% of the participants were cognitively impaired [9].

In addition to the non-infectious risk factors for cognitive impairment that were documented among the elderly in Cameroon (i.e., age, gender, lack of formal education, and higher systolic blood pressure) [8], infection with Human Immunodeficiency Virus (HIV) is also associated with poorer cognitive outcomes in the Cameroonian setting [10,11,12,13,14,15]. Evidence from a multi-cohort study suggests that infection is associated with a one- to five-fold increase in the risk of dementia, and this risk does not vary greatly by the type of infection [16]. Furthermore, studies along the Mbam and Sanaga river valleys in Cameroon have recently reported an association between exposure to the parasite *Onchocerca volvulus* (causative agent of onchocerciasis) and neurological disorders such as CI and epilepsy [17,18,19]. Hitherto, the filarial worm *O. volvulus* was known to cause only skin and eye lesions [20]; however, increasing evidence suggests that it also induces neurological disease [21,22,23]. A recent study in Cameroon found that school-aged children who had been exposed to *O. volvulus* (using Ov16 IgG seropositivity as a proxy) had poorer cognitive performance compared to their unexposed peers from the same community [18]. Until now, no similar explorations have been done among the elderly population, who are more prone to cognitive impairment or dementia on account of their age. Of note, onchocerciasis currently affects an estimated 21 million people worldwide, with 99% of infected persons residing in SSA [20]. Onchocerciasis is the second leading cause of infectious blindness and has been marked for elimination by the year 2030 [24].

This study’s main goal was to evaluate the cognitive performance of elderly persons residing in an onchocerciasis-prevalent area in Cameroon and screen them for dementia to fill the gap regarding dementia epidemiology in the Cameroonian setting. Furthermore, the cognitive findings were compared across elderly persons with and without exposure to *O. volvulus* infection.

## 2. Materials and Methods

### 2.1. Study Design and Setting

Between March and May 2023, a community-based, cross-sectional, and analytical study was conducted in four rural villages of the Ntui Health District of Cameroon, namely: Nachtigal, Ehondo, Ndjame, and Essougli. The study area is found in the Mbam et Kim Division of the Centre Region of Cameroon, located about 70 Km north of Yaoundé, the capital city. The Ntui area is home to an estimated population of 25,615 individuals. Owing to the frequent rural exodus of the youthful population who seek better educational, social, and financial opportunities in neighboring urban communities, the proportion of elderly persons in Ntui has been trending upward in recent years. Agriculture is the main activity in the area, with the production of both food and cash crops. The selected villages are located close to the Sanaga River, which hosts numerous breeding sites for blackflies [25]. Previous parasitological surveys have confirmed the area as being endemic for *O. volvulus* infection [25,26,27], with the most recent data revealing an onchocerciasis prevalence of 14.6% in the Ntui Health District [25]. Furthermore, the Ntui area is also co-endemic for other filarial parasites, notably *Loa loa* and *Mansonella perstans*, albeit having a much lower prevalence compared to onchocerciasis [26].

### 2.2. Study Population and Sampling

This study included all consenting persons aged 60 years and above who had been residing in the study area for at least 12 months. All individuals who were unable to talk fluently or understand the tool used for cognitive assessment were excluded. A consecutive sampling approach was adopted, whereby all persons meeting the inclusion criteria were invited to participate in the study. The goal was to be exhaustive in our sampling by retracing about 150 elderly individuals aged ≥60 years who were identified during a prior door-to-door survey in four study villages: Nachtigal, Ndjame, Ndowe, and Essougli [28]. Given that Ndowe Village was not accessible during our study due to bad roads, it was replaced with the neighboring Ehondo Village.

### 2.3. Data Collection Tools

#### 2.3.1. Cognitive Assessment

To investigate the cognitive performance of elderly persons in rural Cameroon, this study required a validated neurocognitive assessment tool that is simple to administer and would not require participants to be literate. The Community Screening Interview for Dementia (CSID) met these criteria. The CSID tool was developed as a screening instrument for dementia for use in cross-cultural studies and non-literate populations [29]. Pilot studies done using the CSID tool demonstrate its adaptability and utility in populations from poor socioeconomic backgrounds [30]. Furthermore, the CSID tool has a good discriminatory ability in screening for dementia amongst uneducated subjects [31]. The CSID tool consists of two components, namely a cognitive test and an informant interview to document the subject’s performance during everyday activities [30]. Although the combination of cognitive and informant CSID scores was shown to achieve better sensitivity and specificity for dementia screening than cognitive testing alone, the fact that many participants in our study did not have reliable informants (spouse or family member capable of answering specific questions) led us to exclude the informant CSID score from our analysis. Based on a community study conducted in Nigeria by Hall et al., which suggested that CSID cognitive scores above 29.5 can be considered as normal [29], our study adopted a CSID cut-off score of ≤29.5 as a positive screening for dementia, since lower scores indicated sub-normal cognitive performance. In addition, all study participants underwent a complete clinical assessment by a neurologist to ascertain the clinical diagnosis.

#### 2.3.2. Sleep Assessment

Alongside neurocognitive testing, the quality of sleep of participants was also investigated as a relevant covariate that can influence the participants’ performance during the cognitive assessment. For this purpose, the most widely used sleep assessment tool—the Pittsburgh Sleep Quality Index (PSQI) [32]—was chosen. The PSQI is a self-rated questionnaire that assesses sleep quality and disturbances over a one-month time interval [33]. The PSQI is scored on its first nine items based on a 0 to 3 Likert scale, whereby a score of 3 denotes the negative extreme of the parameter being assessed [33]; the tenth item of the PSQI is not included in the scoring. The scored PSQI items are then regrouped in a pre-specified manner to generate seven “component” scores (subjective sleep quality, sleep latency, sleep duration, sleep efficiency, sleep disturbances, use of sleeping medication, and daytime dysfunction). The sum of the scores for these seven components yields one global score for each participant (range 0–21). Scores from ≤ 5 indicate “good” sleep, and higher scores indicate worse sleep quality.

### 2.4. Study Procedures

Before initiating participant recruitment, the research team (consisting of a neurologist (MKM), a medical doctor (KGN), a biomedical engineer (MAFT), and a research assistant (JNTN)) met with the administrative authorities of each village to discuss the project with them and secure their collaboration. A community liaison agent was identified in each village and was tasked with conducting sensitization and mobilization activities for the village residents to be informed about the study.

The fieldwork for our study unfolded in two phases. In the first phase, the research team embarked on a door-to-door survey with the assistance of the community liaison agent to map out the target population of persons aged 60 years and above in each village. Geographic coordinates were recorded for every home with eligible persons to locate them with ease during the subsequent recruitment phase. This exercise was carried out in the month of March 2023, one month prior to participant recruitment into the study. At the end of the first phase, the research team had a list of potential study participants in the target villages. The second phase consisted of participant recruitment and data collection, which took place between April and May 2023. During this second phase, all eligible village residents who were previously identified were approached, and those who consented to participate were enrolled in the study.

At each research site, three stations were set up to facilitate the enrollment of participants and data/sample collection during the second phase of the fieldwork. Station 1 (operated by the principal investigator) was the first point of contact with the participants, where the latter were informed about the study to obtain their consent to participate. This station also recorded all the socio-demographic data, blood pressure, and anthropometric measurements (weight and height) of the participants. The visual acuity of the participants was assessed using the Snellen chart [34] and scored from zero to ten before they were sent to the next station. At Station 2, the neurologist examined the participants and evaluated their cognitive performance using the CSID tool in the French language (understood by all participants). Sleep quality was assessed by the neurologist using the PSQI tool. Finally, the participants progressed to Station 3 (laboratory), where two skin snips were aseptically obtained from their lower back (one from each side, around the iliac crests) for the onchocerciasis diagnosis. The skin snips were incubated in saline for 24 h; after which, the biomedical engineer counted the number of *O. volvulus* microfilariae in each skin snip under a light microscope. Based on the availability of the consumables, some participants also benefited from onchocerciasis Ov16 IgG antibodies serological testing using a rapid diagnostic test (RDT) (DDTD Biplex prototype C, San Diego, CA, USA). Antibody testing with the RDT was performed as per the manufacturer’s instructions, using a drop of blood obtained by finger-pricking the participants.

### 2.5. Data Management and Analysis

All collected data were entered into Microsoft Excel 2019 spreadsheets for cleaning and transferred to R version 4.3.3 for analysis. The CSID scores were dichotomized using the cut-off ≤29.5 to classify participants as having dementia [29]. In a like manner, the PSQI scores were dichotomized to classify participants as having good sleep (score ≤ 5) vs. poor sleep quality (score > 5). For an onchocerciasis diagnosis, the skin snip results were interpreted as positive or negative depending on whether *O. volvulus* microfilariae were detected during the microscopic examination of the sample post-incubation, and the mean microfilarial loads were calculated. For the descriptive analyses, the Ov16 RDT results were also used as a proxy for participants’ exposure to *O. volvulus*. Continuous variables were summarized as the mean with standard deviation and compared across groups using parametric or non-parametric tests as appropriate. Categorical variables were expressed as counts and percentages and compared using the chi-square test or Fisher’s exact test as appropriate.

A multivariable linear regression model was constructed using the CSID cognitive scores as the dependent variable and onchocerciasis infection status (by skin snips) as an independent variable, adjusted for age, sex, education level, and any other covariates with *p* < 0.2 in the univariate analysis. A significance threshold of 5% was adopted for all analyses.

### 2.6. Ethical Considerations

Our study protocol was approved by the institutional review board of the Faculty of Medicine and Biomedical Sciences of the University of Yaoundé I, Cameroon (Ref. No. 861/UYI/FMBS/VDRC/DAASR/CSD on 9 January 2023). An administrative research permit to conduct fieldwork in the study area was granted to the senior author (AKN) by the Ministry of Scientific Research and Innovation of Cameroon (Ref: 000144/MINRESI/B00/C00/C10/C13). All study procedures were in conformity with the Declaration of Helsinki and Good Clinical Practices. Informed consent was obtained from all study participants. All collected data were treated with absolute confidentiality.

## 3. Results

### 3.1. Participant Characteristics

Overall, 103 elderly participants were recruited (mean age: 67.0 ± 6.1 years; range: 60–87 years). There were 49 (47.6%) male participants and 54 (52.4%) female participants. Nachtigal Village contributed the highest number of participants (*n* = 39, representing 37.9% of the study population). The socio-demographic characteristics and relevant medical features of the participants are summarized in Table 1.

### 3.2. Clinical Findings in Participants

#### 3.2.1. Overall Clinical Findings

Out of the total participants, 29 (28.2%) had a body mass index (BMI) greater than 25, while only 5 (4.8%) had a BMI greater than 30. Even though 72.8% of the participants reported experiencing skin itching, only 13 (12.6%) of them had palpable subcutaneous nodules. The prevalence of *O. volvulus* infection was 17.9% based on the skin snip microscopy results (Table 2). Out of the 95 participants that had skin snip and/or Ov16 serological data available, 46 (48.4%) tested positive for at least one of the tests. Furthermore, 7/91 (7.7%) of participants with available data on visual acuity had a Snellen’s score of zero, of which 6 (6.6%) were blind in both eyes.

#### 3.2.2. Prevalence of Dementia

**Figure 1:**
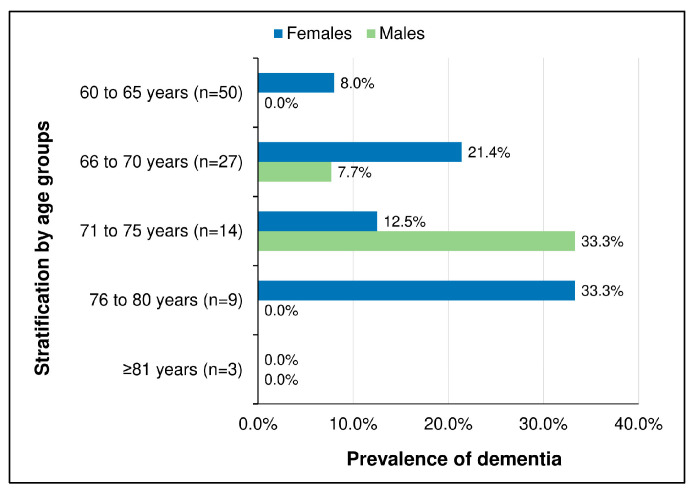
Prevalence of dementia by age and by sex.

Based on the ≤29.5 cut-off of the CSID tool, the prevalence of dementia in our study population was 10.7% (11 out of 103 participants). The seven visually impaired participants all had missing data for items 2–8, 29, and 31 of the CSID tool, as these required visual capacities. Despite this, only one of them scored below the cut-off of 29.5. Although the mean CSID scores were higher among males (40.7 ± 6.5) compared to females (35.7 ± 8.0; *p* = 0.001), the sex-specific prevalence of dementia was not significantly different between sexes: 3/49 (6.1%) for males vs. 8/54 (14.8%) for females; *p* = 0.154. Meanwhile, there was a trend of increasing dementia prevalence across age groups (*p* = 0.057). However, none of the three participants aged above 80 years screened positive for dementia (see Figure 1).

**Figure 2:**
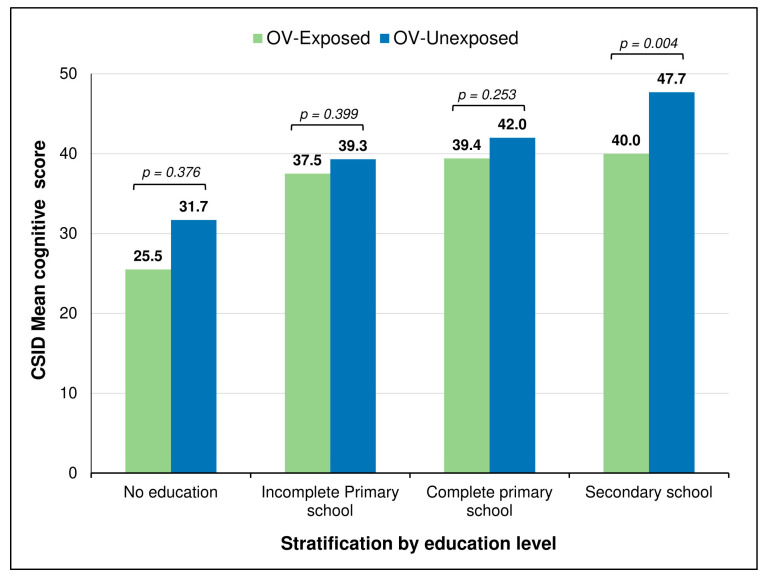
Mean cognitive scores by onchocerciasis exposure and education level. CSID: Community Screening Interview for Dementia; OV: *Onchocerca volvulus*.

The participants’ cognitive scores were also analyzed based on their onchocerciasis infection status, and this revealed a trend of lower scores among onchocerciasis-exposed individuals, but the difference was not statistically significant (Table 3). However, upon stratifying the data by education level, it revealed that participants who had been exposed to *O. volvulus* (positive skin snip and/or Ov16 serology) had lower mean CSID scores compared to their unexposed counterparts (Figure 2).

### 3.3. Factors Associated with CSID Cognitive Score

The linear regression showed that the following covariates were associated with increased cognitive scores: male sex, higher education level, and better visual acuity (Table 4). Meanwhile, inhaling tobacco products (“snuff”) was associated with significantly lower CSID scores. Furthermore, a borderline association was observed with the “positive skin snip” variable (*p* = 0.055), whereby having *O. volvulus* microfilaria (positive skin snips) tended to reduce the participants’ cognitive performance, as measured by the CSID tool.

## 4. Discussion

This is the first study of an association between dementia and *Onchocerca volvulus*, to the best of our knowledge. Our community-based approach using the CSID tool has yielded a prevalence of dementia of 10.7% in the Ntui Health District of Cameroon. Male participants were more likely to score higher on the CSID, concurring with previous data reporting a higher prevalence of dementia among females, possibly due to their longer life expectancy [35]. The 10.7% prevalence value in our study is more representative of the epidemiology of dementia in rural Cameroon in view of the rural adequacy of the tool used. Furthermore, a recent study in rural Kenya, using the brief CSID and applying a “method of back estimating screen positives based on established sensitivity and specificity of the tool against a gold standard”, reported a dementia prevalence of 9.4% (95% CI: 8–11%) [36], which is close to our result. Another recent paper has compared the performance of CSID and other screening instruments and found it valid for a multicultural and multiethnic Asian population [37]. Our community-based approach gives a better prevalence estimate for dementia in Cameroon compared to studies conducted in the hospital setting. Indeed, previous hospital-based studies that reported dementia in elderly Cameroonians already suffering from other ailments tended to overestimate the prevalence of dementia, notably at 11.1% among hypertensive patients in Yaoundé [38], 12.4% among neurological patients at a clinic in Yaoundé [39], 19% among people with degenerative disorders in Yaoundé [40], and 21–59% among persons living with HIV in Yaoundé and Bamenda [10,41]. Also, the fact that our study utilized the CSID tool for dementia screening in rural Cameroon most likely yielded more reliable results compared to studies that employed other tools, such as the Montreal Cognitive Assessment (MoCA) and the Mini-Mental State Examination (MMSE), which underestimate the influence of education level when screening for dementia [42].

As expected, higher education levels resulted in increased CSID scores and vice versa. When stratified by educational level, onchocerciasis-exposed participants scored lower on the CSID tool, suggesting a poorer cognitive performance in this group. This observation was confirmed by the regression analysis, which showed a borderline association between being infected with *O. volvulus* and scoring, on average, 3.4 points lower on the CSID; we surmise that using more sensitive *O. volvulus* detection approaches such as PCR for detecting microfilarial DNA or LIPS (luciferase immunoprecipitation system assay) for detecting antibodies to host neuronal proteins [43] might have made this association statistically significant. While this is the first report describing a potentially negative influence of onchocerciasis on the cognitive performance of the elderly, a similar pattern had been observed by our group among school-aged Cameroonian children, whereby Ov16 seropositivity (as a proxy for onchocerciasis exposure) was significantly associated with lower neurocognitive scores [18]. Other infections, such as HIV and malaria, have also been associated with poorer cognitive outcomes in the Cameroonian setting [10,11,41,44]. It is becoming increasingly evident that infections constitute an important risk factor for dementia [16]; this is not restricted to infections of the central nervous system but includes other infections that can elicit a systemic response. Therefore, it would be worthwhile for future research to investigate the pathogenic mechanisms by which *O. volvulus* infection could impact brain health, resulting in poorer neurocognitive performance.

Onchocerciasis prevalence by skin snip microscopy was 17.9% in our study population; additionally, more than 60% of tested participants were Ov16-seropositive, indicating previous exposure to *O. volvulus.* From our data, it is unclear whether the association between higher visual acuity and higher CSID scores is due to the fact that persons with poor vision are more likely to be *O. volvulus*-infected or because the visual impairment limited their ability to optimally execute some of the tasks included in the CSID tool, such as identifying objects/body parts or drawing on a sheet of paper. This warrants further investigation. That notwithstanding, positive skin snip and Ov16 results reveal that onchocerciasis transmission is still ongoing in the Ntui Health District despite decades of mass treatment with ivermectin [25]. Therefore, the onchocerciasis elimination program needs to be strengthened by improving the annual ivermectin coverage and by considering alternative strategies, such as community-based vector control using the “Slash and Clear” method, which is feasible in such settings [45,46,47].

In line with findings from a large population-based longitudinal study in LMICs [48], cigarette smoking was not significantly associated with cognition in our study. Meanwhile, the regression model showed that inhaling tobacco (snuff) was a significant predictor for lower CSID scores. A study in Zambia found similar results, with poorer neurocognitive outcomes (specifically pertaining to attention and working memory) among women who consumed snuff more frequently; of note, the most common route of snuff consumption in that study was nasal inhalation [49]. This finding contradicts the common thought that smokeless tobacco products are relatively harmless. Indeed, beyond inducing cognitive problems, there is overwhelming evidence incriminating smokeless tobacco products in cancers and coronary heart disease [50]. Long-term inhalation of snuff is also detrimental to the nasal mucosa, as it favors the development of a form of chronic rhinitis [51]. There is a need for sensitization and policy shifts to minimize the usage of snuff and other smokeless tobacco products by the public in view of their overlooked health consequences [52]. Public health stakeholders are called upon to act rapidly against the use of smokeless tobacco products among all population groups so as to avoid the adverse health consequences of these substances, which are erroneously labeled as “harmless” by many.

We did not find any significant association between sleep quality and the CSID scores, whereas poor sleep has been tagged as a risk factor for cognitive decline and, consequently, dementia [53]. The sleep measures in our study were more subjective. Therefore, additional research on sleep using more objective tools and larger sample sizes is necessary to further investigate this lack of association in this setting.

Overall, our results respond to the call of the Nairobi Declaration [7] by providing the prevalence and possible environmental and behavioral risk factors for dementia in rural Cameroon. It is expected that this evidence will inform national brain health policy, within a broader framework of the national disease control programs (integrating both non-communicable disease control and communicable disease programs), building on the well-established national control programs such as for diabetes and epilepsy [54] and aligning with the WHO’s Intersectoral Global Action Plan for epilepsy and other neurological diseases [55].

### Study Limitations

Our study had a few limitations, notably the small sample size and the fact that not all participants were tested for onchocerciasis. Also, the cross-sectional design of our study does not permit us to infer causality between *O. volvulus* infection and dementia but merely suspects an association that should be investigated in larger prospective studies. Additionally, the timeline of the participants’ exposure to onchocerciasis is unknown, and therefore, in case *O. volvulus* is indeed detrimental to the cognitive wellbeing of elderly persons, the duration of exposure that would result in clinically relevant changes remains unclear. We did not investigate the ivermectin intake history of the participants, as this drug can significantly alter the skin snip results in cases of recent administration. However, this is expected to affect our findings only marginally, because the previous ivermectin mass drug administration campaign in the study area was done in July 2022, more than eight months before our participants were recruited. Finally, we acknowledge that our study procedures were far from exhaustive in identifying possible infectious causes of cognitive decline among the participants, as no investigations were done to exclude neurological disease caused by HIV, toxoplasmosis, or neurocysticercosis. Larger studies with a more comprehensive assessment of the participants are required to further explore the possible association between *O. volvulus* infection and cognitive performance in the elderly.

## 5. Conclusions

Using a community-based approach and the validated CSID tool for cognitive evaluation, our study has provided reliable data on the prevalence of dementia in rural Cameroon, estimated at 10.7%. A borderline association between lower CSID scores and *O. volvulus* infection (detected by microscopy) suggests that this parasite may induce neurocognitive symptoms, as previously suspected, with epilepsy. Strengthening onchocerciasis elimination, as well as other communicable and non-communicable disease control programs and adopting healthier lifestyles (for instance, by quitting snuff consumption), would contribute to decreasing the incidence of dementia among the elderly in such settings.

## Figures and Tables

**Table 1:** Characteristics of the study participants.

Characteristics	Findings
All participants: *n* (%)	103 (100%)
Village: *n* (%)	
Ehondo	7 (6.8%)
Essougli	30 (29.1%)
Nachtigal	39 (37.9%)
Ndjame	27 (26.2%)
Age: mean (standard deviation)	67.0 (6.1)
Sex: *n* (%)	
Female	54 (52.4%)
Male	49 (47.6%)
Profession: *n* (%)	
Farmer	94 (91.3%)
Housewife	5 (4.85%)
Business	2 (1.9%)
Construction	2 (1.9%)
Education level: *n* (%)	
None	13 (12.6%)
Primary, incomplete	60 (58.3%)
Primary, completed	23 (22.3%)
Secondary	7 (6.8%)
Marital status: *n* (%)	
Married	60 (58.3%)
Divorced/Widowed	38 (36.9%)
Single	5 (4.9%)
History of alcohol consumption: *n* (%)	79 (76.7%)
History of tobacco consumption: *n* (%)	
Smoking cigarettes	13 (12.6%)
Inhaling tobacco powder	17 (16.5%)
None of the above	73 (70.9%)
History of illicit drugs use: *n* (%)	0 (0%)
Known to have hypertension: *n* (%)	27 (26.2%)
Known to have epilepsy: *n* (%)	0 (0%)
Known to have diabetes: *n* (%)	0 (0%)
Known history of stroke: *n* (%)	2 (1.9%)

**Table 2:** Clinical findings in the enrolled participants.

Clinical Characteristics	Findings	N *
Body Mass Index: mean (SD)	22.7 (4.4)	103
Systolic blood pressure: mean (SD)	139 (26.3)	103
Diastolic blood pressure: mean (SD)	82.1 (17.4)	103
Palpable nodules: *n* (%)	13 (12.6%)	103
Itching: *n* (%)	75 (72.8%)	103
Depigmentation: *n* (%)	5 (4.9%)	103
Snellen’s score for visual acuity: mean (SD)	5.67 (2.8)	91
Sleep quality		84
Good Sleep, PSQI score ≤ 5: *n* (%)	54 (64.3%)	
Poor Sleep, PSQI score > 5: *n* (%)	30 (35.7%)	
Positive skin snips: *n* (%)	15 (17.9%)	84
Microfilarial load in positive skin snips: Mean (SD)	14.1 (19.6)	15
Ov16 seropositivity: *n* (%)	41 (62.1%)	66

* *n* = number of participants with available information on the variable of interest, excluding missing data. SD: Standard deviation; PSQI: Pittsburgh Sleep Quality Index.

**Table 3:** Onchocerciasis exposure and dementia among study participants.

	Exposed	Unexposed	*p*-Value	*n*
*Using skin snip positivity only for onchocerciasis exposure*				
Participants with dementia (CSID ≤ 29.5): *n* (%)	3/15 (20.0%)	6/69 (8.7%)	0.198	84
Participants without dementia (CSID > 29.5): *n* (%)	12/15 (80.0%)	63/69 (91.3%)		
CSID score: Mean (Standard Deviation)	36.2 (8.0)	38.6 (7.6%)	0.303	84
*Using Ov16 seropositivity only for onchocerciasis exposure*				
Participants with dementia (CSID ≤ 29.5): *n* (%)	5/41 (12.2%)	1/25 (4.0%)	0.396	66
Participants without dementia (CSID > 29.5): *n* (%)	36/41 (87.8%)	24/25 (96.0%)		
CSID score: Mean (Standard Deviation)	37.7 (7.8)	40.0 (7.8)	0.259	66
*Using skin snips positivity and/or Ov16 serolopositivity for onchocerciasis exposure*				
Participants with dementia (CSID ≤ 29.5): *n* (%)	6/46 (13.0%)	4/49 (8.2%)	0.516	95
Participants without dementia (CSID > 29.5): *n* (%)	40/46 (87.0%)	45/49 (91.8%)		
CSID score: Mean (Standard Deviation)	37.6 (7.7)	39.3 (7.9)	0.289	95

CSID: Community Screening Interview for Dementia.

**Table 4:** Linear regression model investigating the factors associated with cognitive score.

Model * Covariates	Odds Ratio (95% CI)	*p-Value*
Positive skin snip	−3.399 (−6.805 to 0.007)	0.055
Age	0.005 (−0.213 to 0.223)	0.964
Male sex	4.775 (1.997 to 7.553)	0.001
Education		
None	Reference	
Primary, incomplete	3.565 (−0.742 to 7.871)	0.109
Primary, complete	4.498 (−0.2153 to 9.212)	0.066
Secondary	8.654 (2.0870 to 15.222)	0.012
Body mass index	0.130 (−0.1434 to 0.404)	0.354
Visual acuity (Snellen score)	1.122 (0.644 to 1.600)	<0.001
Poor sleep quality (PQSI > 5)	1.828 (−0.828 to 4.485)	0.182
Alcohol consumption	0.369 (−2.910 to 3.647)	0.826
Tobacco consumption		
No	Reference	
Inhaled tobacco	−5.441 (−9.137 to −1.744)	0.005
Cigarettes	−0.596 (−4.5005 to 3.308)	0.766

* *n* = 80 (23 participants with missing data omitted); Pseudo-R² (Cragg-Uhler) = 0.56; AIC = 515.7.

## Data Availability

The data presented in this paper are freely available from the corresponding author upon reasonable request.
